# Editorial: Athlete vulnerabilities and doping

**DOI:** 10.3389/fspor.2024.1378777

**Published:** 2024-02-16

**Authors:** Kim Nolte, Angela Jo-Anne Schneider

**Affiliations:** ^1^ Division of Biokinetics and Sport Science, Department of Physiology, University of Pretoria, Pretoria, South Africa; ^2^ School of Kinesiology, International Centre for Olympic Studies, Western University, London, ON, Canada

**Keywords:** doping, vulnerability, sport, values, supplements

**Editorial on the Research Topic** Athlete vulnerabilities and doping

Although society tends to embrace performance enhancement in various aspects of life, particularly within the domains of work and physical attributes that are commonly associated with beauty, the ethical objections raised in these two realms, do not compare to the frequency and intensity that arise in the realm of enhancement in sport—doping. Since sport functions as a sort of paradigm for society, there is an anticipation of elevated norms of fairness and equal opportunities for participants within it. Doping is a complex problem and threatens the integrity of sport (1). The bans on doping stem from a shared commitment to fostering enduring human excellence, acknowledging that success in sports should harmonize with the broader goal of promoting lifelong well-being. No single factor can explain doping but rather a host of interrelated factors. There are serious consequences if an athlete is found guilty of doping. Detrimental consequences and testing alone will not solve doping in sport. In order to overcome this ever-increasing problem, it is necessary to conduct research and implement various strategies aimed at decreasing the likelihood of athletes doping. An understanding of which factors influence an athlete's vulnerability to doping can potentially provide important information for the implementation of interventions to decrease doping. This understanding can also help to protect athletes as the sport context increases athlete vulnerability in general and even more so for doping in particular. The concept of vulnerability itself is an understudied concept with great significance to our understanding of the human condition. Thus, the primary aim of this Research Topic was to gather research in the field of doping in sport, particularly regarding athlete vulnerabilities and doping (inadvertent or intentional).

This Research Topic comprises of six articles. It is not surprising that two of the articles relate to supplement use. Athlete supplement use has been regarded as a doping “gateway”. Athlete's supplement consumption may increase doping risks through the development and acceptance of routine substance use to improve sporting performance (2, 3). Furthermore, supplement use in athletes can also result in inadvertent doping due to supplement contamination or the uneducated use of supplements that contain prohibited substances. In the review by Yasuda et al. the authors summarize how the “meal first” strategy and planned supplement use are important for enhancing athletes’ health and performance. The “meal first” strategy is recommended for athletes’ conditioning, however there are a few instances when appropriate supplement use can be beneficial to athletes such in the case of nutrient deficiencies, inaccessibility of quality food during travel and difficulty preparing food due to societal restrictions, to mention a few (Yasuda et al.). In a quantitative analysis of factors which influence supplement use and doping among adolescent athletes in New Zealand, Clancy et al., identified factors that decrease adolescent's chances of doping. To reduce the risk of doping, the researchers suggest attention should be given to increasing athlete's exposure to mastery as a confidence source, reducing focus on appearance and supporting volition/internally perceived locus of control (Clancy et al.).

When it comes to vulnerability to doping, vulnerability is not constant and can change during the athlete's sporting career or athlete pathway. Thus, it is also important to understand how vulnerability may change, and thus, when specific interventions may be necessary or beneficial to decrease vulnerability and potential doping violations. The results of the research by Veltmaat et al., showed that athletes believed that “clean” and “doping” athletes are not always distinguished by the values they hold. Therefore, all athletes can be vulnerable to doping at some point. Further strengthening the point of view that vulnerability is not constant. The researchers concluded that vulnerability is a balance between risks and protective factors in a complex interaction between environmental, personal and situational influences (Veltmaat et al.).

Educational interventions are another area of focus of the World Anti-doping Agency (WADA). Some research has shown that many athletes do not have sufficient knowledge regarding doping/anti-doping, which has significant consequences given the “strict liability” rules. Lack of knowledge may then lead to possible doping violations. Murofushi et al. investigated subjective and objective anti-doping knowledge in Japanese University students and found that there was a discrepancy between subjective and objective anti-doping knowledge. Athletes perceived themselves to have adequate anti-doping knowledge however objective measures indicated otherwise. The researchers suggest targeted educational interventions to align subjective and objective anti-doping knowledge. Even if athletes recognize doping as wrongful, insufficient knowledge may inadvertently influence behavioral intentions, potentially leading to unintentional doping. The researchers also investigated the willingness to learn and objective anti-doping knowledge in the same cohort (Japanese University students) and found no relationship between willingness to learn and objective anti-doping knowledge. Although no relationship was determined in this study, the results did show that the athletes had insufficient knowledge regarding doping and that educational interventions were warranted (Murofushi et al.).

Lastly, an article by Pavot further highlights the challenges and complex struggle to combat doping in sport and to ensure all athletes are able to compete within a fair environment. The article discusses the potential gap or lacuna in the World Anti-Doping Code (WADC) considering the case of the Russian figure skater—Kamila Valieva. The article concluded that the identification of the gap and the interpretation of the rules by the Court of Arbitration for Sport (CAS) was questionable in this award (Pavot). This case highlights athlete's vulnerability in the sense that they depend on the proper implementation of anti-doping rules and regulations so that they can compete fairly. Athletes must be protected from an opponent who has potentially cheated.

From the research included in this collection, the following conclusions can be drawn. Vulnerability is not constant, and vulnerability may change during the athlete's career. It is necessary to consider the environmental, personal and situational influences and to try to balance risks and protective factors. Supplement use remains an important factor that can increase the vulnerability of the athlete doping. Interventions supporting volition or internally perceived locus of control and increasing athlete's exposure to mastery as a confidence source may be beneficial. However, it is also important to note that in some circumstances supplementation may be beneficial to the athlete and therefore in these situations the necessary education and guidance would be invaluable as athletes too have a right to a standard of care for their health and wellness. Specific cohorts of athletes lack sufficient knowledge regarding anti-doping and therefore educational interventions remain pivotal. Finally, athletes remain vulnerable to the systems and regulations in place to protect them and to ensure they compete within a fair environment.
